# Influence of the Soil Genesis on Physical and Mechanical Properties

**DOI:** 10.1155/2013/454710

**Published:** 2013-06-10

**Authors:** Marian Marschalko, Işık Yilmaz, Lucie Fojtová, Karel Kubečka, Tomáš Bouchal, Martin Bednárik

**Affiliations:** ^1^Faculty of Mining and Geology, Institute of Geological Engineering, VŠB-Technical University of Ostrava, 17 Listopadu 15, 708 33 Ostrava, Czech Republic; ^2^Department of Geological Engineering, Faculty of Engineering, Cumhuriyet University, 58140 Sivas, Turkey; ^3^Department of Building Structures, Faculty of Civil Engineering, VŠB-Technical University of Ostrava, 17 Listopadu 15, 708 33 Ostrava, Czech Republic; ^4^Faculty of Mining and Geology, Institute of Environmental Engineering, VŠB-Technical University of Ostrava, 17 Listopadu 15, 708 33 Ostrava, Czech Republic; ^5^Department of Engineering Geology, Faculty of Natural Sciences, Comenius University, Mlynská dolina, 842 15 Bratislava, Slovakia

## Abstract

The paper deals with the influence of soil genesis on the physical-mechanical properties. The presented case study was conducted in the region of the Ostrava Basin where there is a varied genetic composition of the Quaternary geological structure on the underlying Neogeneous sediments which are sediments of analogous granulometry but different genesis. In this study, 7827 soil samples of an eolian, fluvial, glacial, and deluvial origin and their laboratory analyses results were used. The study identified different values in certain cases, mostly in coarser-grained foundation soils, such as sandy loam S4 (MS) and clayey sand F4 (CS). The soils of the fluvial origin manifest different values than other genetic types. Next, based on regression analyses, dependence was proved neither on the deposition depth (depth of samples) nor from the point of view of the individual foundation soil classes or the genetic types. The contribution of the paper is to point at the influence of genesis on the foundation soil properties so that engineering geologists and geotechnicians pay more attention to the genesis during engineering-geological and geotechnical investigations.

## 1. Introduction

Many buildings are constructed with foundations that are inadequate for the soil conditions existing on the site. Because of the lack of geographically convenient suitable land, homes are often built on marginal land that has insufficient bearing capacity to support the substantial weight of a structure. Land becomes scarce with the growth of cities, and it often becomes necessary to construct buildings and other structures on the sites with unfavourable conditions [1–6]. That is why generally some detailed researches are needed for soil foundations. An important factor for building foundations is the particle-size distribution of foundation soils and their geotechnical properties. The origin of the foundation soils should also be taken into consideration as on the grounds of good command of the issue it is possible to expect a certain behaviour of the given sediment type already during engineering-geological and geotechnical investigations. Foundation soils of various types and their ages are very heterogeneous, in many cases nonuniform and insufficiently explored deeper. 

Geotechnical characteristics of different types of foundation soils were dealt with by Franks and Woods [7] and Pfleiderer et al. [8], of soft or more precisely muddy soils by Que et al. [9], covering sediments by Khadge [10], lodgement tills by Little and Atkinson [11], of marine sediments by Lee et al. [12], and so forth. As for some other studies on soil genesis, soil genesis and reforestation success were evaluated by Miller et al. [13], and Brevik [14] investigated soil properties in a sand borrow pit in Southern Georgia (USA) that was used in 1961 and then abandoned with no efforts at reclamation and compares those developing soils to natural soils surrounding the pit, and so forth. 

This study describes the geotechnical properties of foundation soils of various genetic types which occur in the area of interest defined by 5 map sheets in 1 : 10 000 scales, with the City of Ostrava and adjacent municipalities, a part of the town of Bohumín in the north-east of the Czech Republic. It is the question of glacial, fluvial, and eolian sediments of a Quaternary age. The produced database of archive data resulted in statistically processed results of laboratory tests, carried out on 7 775 soil samples, determining the physical and mechanical properties. The aim is to evaluate to what extent the individual foundation soil properties vary in dependence on their origin. Consequently, the effects of the sediment deposition on its geotechnical properties were evaluated by means of a regression analysis.

Unlike other works ([8, 15, 16], etc.), the presented study brings an evaluation of a far wider set of foundation soil samples than common in the professional literature. This has been motivated by the existence and availability of a quantity of archived engineering-geological and geotechnical investigations with conducted laboratory and field tests of soils and rocks in the Czech Republic, or other countries, while they have not been processed from the point of view of regional and scientific studies. The archived investigation results are used only in individual surveys of engineering work, such as studies of local exploration for correlation with newly implemented exploration work and tests. It is a shame not to use such data as the test results are only mentioned in the investigation final reports, and there is no database of foundation soil properties. Still, the database itself is not a sufficient tool to evaluate suitability or unsuitability of the foundation soils by means of their geotechnical characteristics, and it is vital to carry out the so-called typology with a follow-up interpretation of local characteristics.

## 2. Stratigraphical and Geological Settings

**Figure 1:**
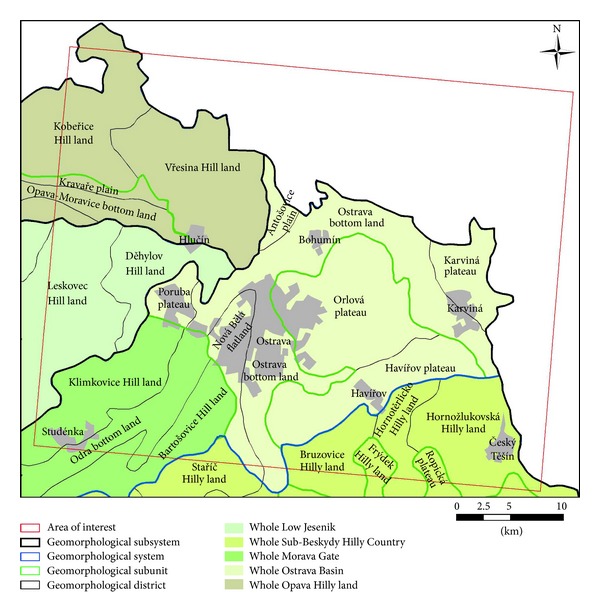
Geomorphological units in the area of interest [17].

As for geomorphology, the area belongs to the Alpine-Himalayan system, subsystem of Carpathians, province of Western Carpathians, and two systems. It is the system of Outer Carpathian Depression which includes the whole of Ostrava Basin in the subsystem of Northern Outer Carpathian Depression and a whole of Morava Gate in the subsystem of Western Outer Carpathian Depression. In the second system of Outer Western Carpathians, there is a subsystem of West-Beskydy Foothills and a whole of Sub-Beskydy Hilly Country. A part of the area also belongs to the Hercynian System and two subsystems. The subsystem of Epihercynian Lowland is further divided into the province of Central-European Lowland, a system of Central Polish Lowlands, a subsystem of Silesian Lowland, and a whole of Opava Hill land. The subsystem of Hercynian Mountain Range also incorporates the province of Czech Highlands, a system of KrkonoŠe-Jesenik, a Jesenik subsystem, and a whole of Low Jesenik (Figure 1) [17]. 

The area of interest is found in the northern part of Western Carpathian Foredeep at the contact of geological units of the Bohemian Massif and the Carpathian System [18]. The subject of the study is the Quaternary sediments which lie on the rocks of the Moravia-Silesian Palaeozoic predominantly represented by Carboniferous rocks and on earlier Neogeneous sediments of the Carpathian Fore deep deposited in the course of marine transgression from Eggenburg to Badenian. Next, the Quaternary sedimentation followed [18]. 

In the Pleistocene, in the age of Elster and Saale glaciations, the continental glacier penetrated the area from the north, followed by depositing of glacial and glaciofluvial poor-graded gravelly and sandy sediments of a brown, grey-brown, yellow, or reddish colour, and glaciolacustrine sediments, represented by grey-reddish, grey-brown, or reddish-brown clays and sandy clays, laminated in grey and with sandy lenticulars in places. During glaciations in the Late Pleistocene, the glacier did not reach as far as the Czech Republic, and mainly eolian and deluvioeolian sediments deposited [19], which are predominant in the area of interest and are represented by loess loam, and lower percentage of sandy clays and loam and sands of yellow-brown and brown to grey-brown colour usually with grey or reddish streaks of predominantly solid consistency and low to intermediate plasticity. 

The Holocene is represented by fluvial sediments of a variable character deposited in the river beds, made up by coarse-grained soils with a minimum share of fine-grained fraction, which on the other hand occurs in overbank sediments in the form of flood loams and alluvial cones. Loamy sediments and unsorted fragments are characteristic of deluvial deposits [19]. 

## 3. Dataset and Methods

**Figure 2:**
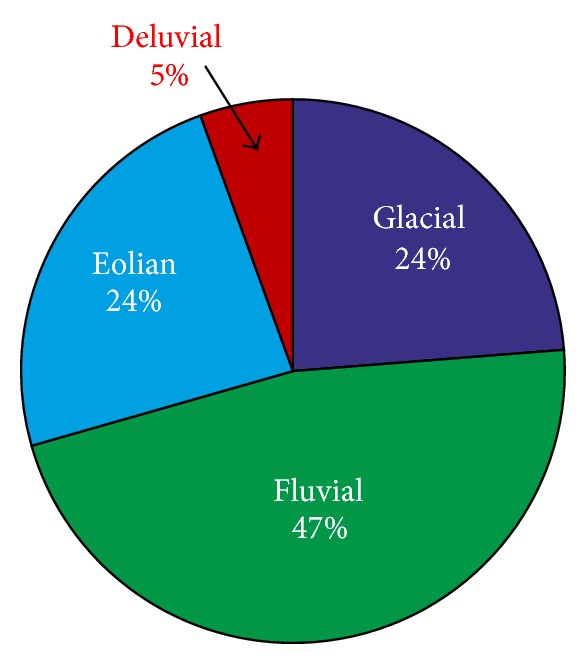
Percentages of the individual genetic types.

**Figure 3:**
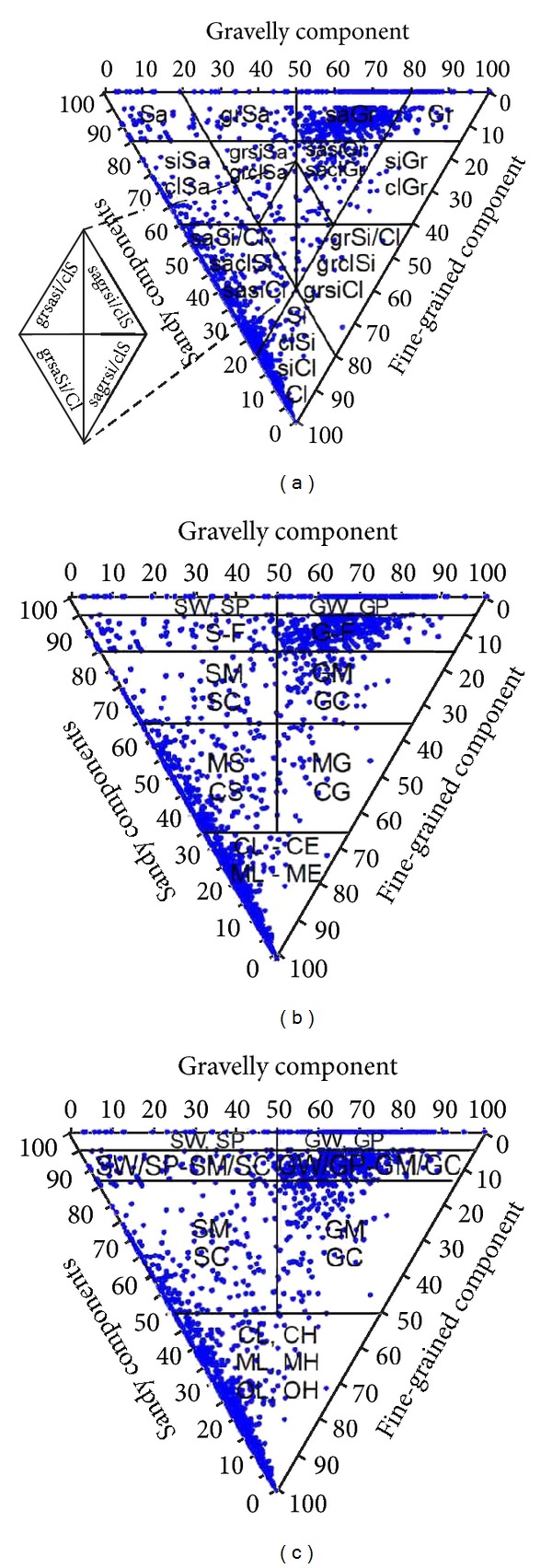
Gr: gravel, Sa: sand, Si: silt, Cl: clay; gr: gravelly, sa: sandy, si: silty, cl: clayey. European, Czech, and USCS classification system of soils with marked studied samples: (a) the European soil classification system [20]; (b) the Czech soil classification system [21], and (c) the Unified Soil Classification System [22].

The engineering-geological characteristics of soil samples, including their spatial orientation, were obtained from archive engineering-geological final reports. Out of the database, compiled selecting required data from the report texts and appendices, containing 6 131 boreholes and 10 658 of drawn soil samples and laboratory analyses results, and 7 827 of studied samples of an eolian, fluvial, glacial, and deluvial origin (Figure 3) were selected. The genetic type is one of the database attributes which is included in the final reports based on the geologists' assistance during drilling work and their knowledge of the geological environment. Therefore, this information may be burdened by an error depending on the individuals' expertise and professionalism (Figure 2).

The most important evaluated properties are density of solid particles (*ρ*_n_), bulk density (*ρ*_s_), dry density (*ρ*_d_), natural water content (w), liquid limit (w_L_), plastic limit (w_P_), plasticity index (I_p_), consistency index (I_c_), porosity (*n*), saturation degree (Sr), effective and total cohesion (c_ef_, c_u_), angle of internal friction (*φ*_ef_, *φ*_u_), and oedometric modulus (*E*_oed_).

**Figure 4:**
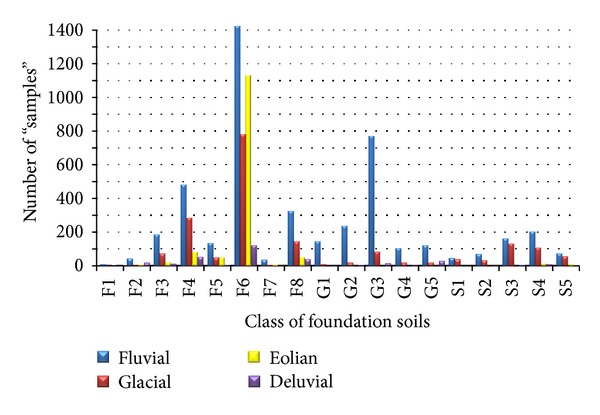
Number of the studied samples of different genetic types in the individual foundation soil classes.

Physical-mechanical properties of soils are studied in terms of particle size distribution, which are represented by foundation soil classes according to the classification in ČSN 73 1001 Standard (Building foundations. Foundation soil under spatial foundations), which is very similar to the Unified Soil Classification System (USCS; ASTM D2487). The basic difference in the applied foundation soil classifications according to the stated systems is clear from Figure 4, which also depicts the studied samples. In the European standard, the boundary between clay and silt is given by the content of clay particles (<0.002 mm), in other classifications by a line A [I_p_ = 0.73(w_L_ − 20)] in the Casagrande's chart, as in USCS. In addition, clays and loams are specified based on liquid limit, where the ČSN Standard states 5 degrees of plasticity: w_L_ > 90% loam of extremely high plasticity, 90–70% of very high plasticity, 70–50% of high plasticity, 50–35% of intermediate plasticity, and <35% of low plasticity, whereas USCS states only two: high plasticity w_L_ > 50% and low plasticity w_L_ < 50%. The European standard defines 4 degrees of plasticity: nonplastic, low, intermediate, and high, but it does not define their values.

**Figure 5:**
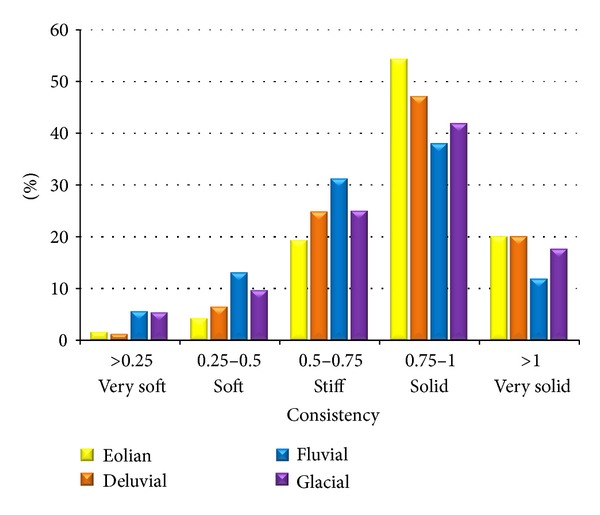
Percentages of the consistency degrees in the individual genetic types.

Based on the content of the individual particle-size fractions determined by the grading curve and the plasticity values (w_L_, w_p_, I_p_), the soil samples were classified into the foundation soil classes. The above mentioned procedure was carried out with 7 775 samples with determined required parameters. The representation of the individual foundation soil classes of various genetic types is in Figure 5. In all the genetic types of the Quaternary age, clays of F6 class (CL, CI) are dominant. Eolian soils have a similar proportion of sandy clays (F4 CS) and clays of high and very high plasticity (F8 CH, CV). Glacial sediments have a lower share of sandy clays of F4 class (CS) than in clays of F8 class (CH, CV) and sands of the classes S3 (S-F) and S4 (SM). There is a similar situation in fluvial and deluvial soils, which are sufficiently represented by coarse-grained soils, mostly by gravels with mixed-in fine-grained soil (G3 G-F) and clayey gravels (CG 5G). It is apparent that eolian sediments are better graded than the other types, in which almost all foundation soil classes are represented.

In order to make a statistic evaluation of the physical-mechanical properties of foundation soils, it was vital to identify the probability distribution of the data set, that is, Gaussian distribution, and it was verified by the Anderson-Darling normality test combined with histograms. Next, outlier values were excluded by means of values outside the lower outlier limits (1)

(1) $$\begin{array}{c} \text{DVH}=Q_{1}-1.5\left(Q_{3}-Q_{1}\right) \end{array}$$

and upper outlier limits (2) of the set (Pavlík, [23])

(2) $$\begin{array}{c} \text{HVH}=Q_{1}+1.5\left(Q_{3}-Q_{1}\right) \end{array}$$

in (1) and (2); *Q*_1_ is the first quartile, and *Q*_3_ is the third quartile. 

Intervals and mean values of physical and mechanical properties were evaluated making use of descriptive statistic characteristics. A regression analysis studied the existence of dependence of physical-mechanical properties on the deposition depth (depth of samples).

A possible occurrence of errors must be taken into consideration, especially gross ones, which may though be simply excluded. This type of errors may have appeared in the laboratory analyses result records of final reports or in the database when copying data. Random errors may have appeared in the geological documentation from the very beginning, when the petrographical description and defined genetic type of soil depended on the geologist's knowledge of geological conditions and experience. Considering the quantity of the processed investigation reports from various companies and thanks to the foundation soil sample analyses carried out according to the national standards as recommended, it may be assumed that any constant errors, methods, or evaluations caused by an identical cause would be apparent. 

## 4. Results

From the point of view of the individual property value intervals, the most varied are fluvial sediments, namely, the class of fine-grained soils and coarse-grained particles and loams, such as gravelly clays (F2 CG), sandy loams (F3 MS), and loams (F5 ML, MI). For example, the water content value interval of class F5 (CL, CI) is 15–29% for eolian foundation soils, 14–53.5% for fluvial foundation soils, and 18–31% for glacial foundation soils, which also manifests in class S4 (SM), and it is 3.5–39% for fluvial foundation soils and 8–24% for glacial ones, and in specific density of gravels with mixed-in clayey soil G3 (G-F) and clayey gravels G5 (GC). To a certain extent, it is influenced by the number of the samples, which increases the value intervals, but the major cause is the poor grading of the sediment given by the character of deposition.

In other cases, there is a difference in the mean values, which is more common in coarse-grained soils. The mean values are important for the specification of an individual property. From the statistic point of view, a median is more important for the data sets as they better describe the range of values. It follows the set being classified into two even parts and is not affected by extreme or outlier values.

Fluvial clayey sands F4 (CS) and sandy loams manifest slightly higher water content values (by 5%) than other genetic types. There is an opposite trend in gravels and sands with mixed-in fine-grained soil G3 (G-F) an S3 (S-F) and loamy sands of class S4 (SM) of the genesis with a difference up to 15%. The porosity of fluvial sandy clays F4 (CS) is also higher than in other genetic types as it reaches as high as 50%. There is a similar situation in class S4 (SM), where fluvial loamy sands reach the maximums of 53% and the glacial of 43%. In the other foundation soil classes, the range or the differences in values are not substantial.

The bulk density is closely connected with the water content and porosity and in class F4 (CS) of a fluvial origin than other foundation soils, whose mean values are 2.07 g·cm^−3^ and 2.09 g·cm^−3^. In case of class S4 (SM), the bulk density of the fluvial type of foundation soils is 1.97 g·cm^−3^ and of the glacial type it is 2.6 g·cm^−3^.

The density of solid particles of fine-grained soils of any genesis ranges from 2.6 g·cm^−3^ to 2.8 g·cm^−3^ with the mean value of 2.69–2.72 g·cm^−3^ for fine-grained soils, with lower values attributed to sandy clays F4 (CS). There is a big difference among clayey gravels G5 (GC) of a fluvial origin, whose density of solid particles in the mean value of 2.69 g·cm^−3^ and of a deluvial origin in the mean value is 2.76 g·cm^−3^. The dry density has a similar trend. Fluvial sandy clays of class F4 (CS) have lower values than other types, that is, 1.32 g·cm^−3^.

The saturation degree is similar in all the cases of mean values of the individual geneses. A difference occurs in the value interval minimums, when, for example, the fluvial and glacial sandy clays F4 (CS) reach the minimums of 0.62 or the eolian and fluvial clays of a low to intermediate plasticity F6 (CL, CI) gain the minimums of 0.7. In coarser-grained foundation soils of a glacial origin in the form of sandy loams F3 (MS) and loamy sands, the mean values of the saturation degree are also analogous, but the minimums start at 0.4.

The consistency states which mainly represent the degree of consistency and the plasticity are evaluated only in fine-grained soils in which there is an apparent, more prominent influence on the mechanical parameters than in coarse-grained foundation soils with minor proportion of fine-grained soils.

**Figure 6:**
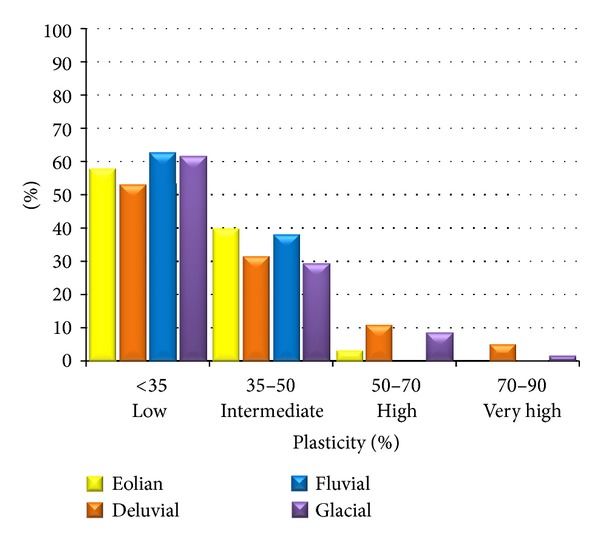
Percentages of the plasticity degrees in the individual genetic types.

As for the consistency of foundation soils of various genetic types, there is no significant difference in the representation of the individual degrees (Figure 6). In all the types, the solid consistency prevails, followed by less stiff and very solid and in a low extent soft and very soft consistency. 

**Figure 7:**
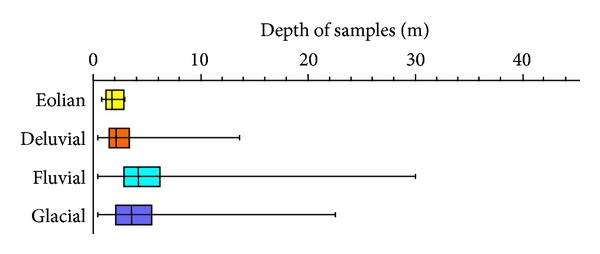
Depth of deposition (depth of samples) of the individual genetic types of foundation soils.

Considering the fact that in fine-grained soils the dominant are clays of low and intermediate plasticity (F6 CL, CI), this plasticity degree also prevails in the overall evaluation of fine-grained foundation soils (Figure 7). High plasticity occurs only in 11% of soils of a deluvial origin, in 8.4% of a glacial origin, and in 3.3% of an eolian origin. Very high plasticity is negligible, namely, in 5.2% of foundation soils of a deluvial origin and in 1.5% of glacial sediments.

Mechanical parameters, such as effective cohesion, the angle of internal friction, and oedometric modulus, were determined with regard to the number of samples divided according to the individual genetic types only for classes F4 (CS), F6 (CL, CI), and F8 (CH, CV). The total shear parameters are measured only in classes F6 (CL, CI) and F8 (CH, CV). As the properties also depend on the consistency, it is convenient to classify them according to this parameter. However, as stated above, the foundation soils are mainly solid and stiff, and a further division still provides a data set of a high number of studied samples, that is, as much as 160. The outcome is that the value intervals as well as the mean values of the properties are very similar or identical. 

**Figure 8:**
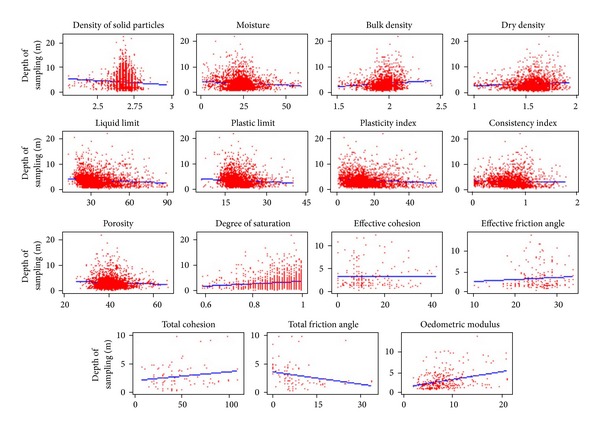
Example dependent of glacial soils on the depth.

Another factor dealt with in the paper is the influence of the foundation soil deposition depth of various genetic types on its properties. This could result in a general principle of a reference hypothesis of the individual property values. Building foundations usually go through various soil depths based on the project or type of construction. Therefore, it is vital to have a basic regional overview of the geological environment. It is clear from Figure 8 that deluvial sediments are found in the mean depth of 2 m and the eolian sediments in the depth of 2.1 m. Fluvial foundation soils reach the mean depth in 3.5 m and glacial in 4.1 m. 

A regression analysis evaluated the existence of dependence between the individual properties of diverse foundation soil classes and different genesis. In any of the attribute combinations, the dependence on the depth was not determined. The coefficient of determination is very low in all the cases, for an example, see Figure 8.

## 5. Conclusions

In conclusion, it may be stated that the influence of various soil type origins, that is, a detailed classification of fine-grained and coarse-grained soils, depends on the good grading and heterogeneity. In case of fluvial and partly glacial sediments, different particle-size distribution growing into high heterogeneity is expected and thus it has diverse properties, such as water content, porosity, bulk density, or density of solid particles only in certain foundation soil classes, namely, fine-grained soils with a percentage of fine-grained particles (F4 CS) or in coarse-grained soils with a high proportion of fine-grained particles (S4 SM). The influence of depth on the geotechnical properties was not established, even despite a sufficient number of the studied samples.

The study identified different values in certain cases, mostly in coarser-grained foundation soils, such as sandy loam S4 (MS) and clayey sand F4 (CS). The soils of the fluvial origin manifest different values than other genetic types. Next, based on regression analyses, dependence was proved neither on the deposition depth (depth of samples) nor from the point of view of the individual foundation soil classes or the genetic types. The contribution of the paper is to point at the influence of genesis on the foundation soil properties so that engineering geologists and geotechnicians should pay more attention to the genesis during engineering-geological and geotechnical investigations.
